# Inhibition of nucleotide biosynthesis disrupts lipid accumulation and adipogenesis

**DOI:** 10.1016/j.jbc.2023.104635

**Published:** 2023-03-23

**Authors:** Abhijit B. Shinde, Elizabeth R. Nunn, Genesis A. Wilson, Mathew T. Chvasta, Julia A. Pinette, Jacob W. Myers, Sun H. Peck, Jessica B. Spinelli, Elma Zaganjor

**Affiliations:** 1 Department of Molecular Physiology and Biophysics, Vanderbilt University, Nashville, Tennessee, USA; 2 Division of Clinical Pharmacology, Department of Medicine, Vanderbilt University Medical Center, Nashville, Tennessee, USA; 3 Department of Veterans Affairs, Nashville Veterans Affairs Medical Center, Nashville, Tennessee, USA; 4 Program in Molecular Medicine, University of Massachusetts Chan Medical School, Worcester, Massachusetts, USA; 5 Vanderbilt Digestive Disease Research Center, Vanderbilt University Medical Center, Nashville, Tennessee, USA; 6 Vanderbilt Diabetes Research Center, Vanderbilt University, Nashville, Tennessee, USA

**Keywords:** lipid droplets, adipocytes, nucleotides, purine, pyrimidine, adipogenesis, metabolism

## Abstract

Energy balance and nutrient availability are key determinants of cellular decisions to remain quiescent, proliferate, or differentiate into a mature cell. After assessing its environmental state, the cell must rewire its metabolism to support distinct cellular outcomes. Mechanistically, how metabolites regulate cell fate decisions is poorly understood. We used adipogenesis as our model system to ascertain the role of metabolism in differentiation. We isolated adipose tissue stromal vascular fraction cells and profiled metabolites before and after adipogenic differentiation to identify metabolic signatures associated with these distinct cellular states. We found that differentiation alters nucleotide accumulation. Furthermore, inhibition of nucleotide biosynthesis prevented lipid storage within adipocytes and downregulated the expression of lipogenic factors. In contrast to proliferating cells, in which mechanistic target of rapamycin complex 1 is activated by purine accumulation, mechanistic target of rapamycin complex 1 signaling was unaffected by purine levels in differentiating adipocytes. Rather, our data indicated that purines regulate transcriptional activators of adipogenesis, peroxisome proliferator–activated receptor γ and CCAAT/enhancer-binding protein α, to promote differentiation. Although *de novo* nucleotide biosynthesis has mainly been studied in proliferation, our study points to its requirement in adipocyte differentiation.

Adipose tissue is a critical organ in coordinating energy balance, releasing nutrients in times of fasting and storing nutrients in times of nutritional excess. In the context of overnutrition, adipose tissue can expand through adipocyte hypertrophy or through the formation of new adipocytes, termed adipogenesis. Adipogenesis is thought to be a protective and adaptive response to excess nutrients. The transcriptional regulation of adipogenesis is well established (1, 2). CCAAT/enhancer-binding proteins (C/EBPs), C/EBPδ and C/EBPβ, are early inducers of adipogenesis (3). These factors stimulate peroxisome proliferator–activated receptor γ (PPARγ), which in turn supports the activation of C/EBPα (4, 5, 6). C/EBPα exerts positive feedback on PPARγ to maintain differentiation. Sterol-regulatory element binding protein 1 (SREBP1) is thought to promote adipogenesis through the production of an endogenous PPARγ ligand and regulation of lipogenic gene expression (7, 8).

Adipogenesis is further modulated through post-translational regulatory mechanisms. In response to nutrients, the mechanistic target of rapamycin complex 1 (mTORC1) stimulates adipogenesis (9). Although the mechanism remains unclear, mTORC1 activity promotes positive feedback between PPARγ and C/EBPα (10, 11). AMP-activated protein kinase (AMPK) is a cellular sensor of energy and nutrient stress and a potent negative regulator of adipogenesis (12, 13, 14). AMPK blocks lipid storage by suppressing lipogenesis while promoting fat oxidation (15). Specifically, activation of AMPK hinders lipogenesis through direct inhibition of SREBP1, which results in significant transcriptional repression of adipogenesis (16). In addition, AMPK antagonizes lipogenesis through the inhibitory phosphorylation of acetyl CoA carboxylase 1 (ACC1). AMPK supports fatty acid oxidation (FAO) through inhibition of acetyl CoA carboxylase 2 (ACC2), which results in decreased malonyl CoA levels and subsequent increased activity of a rate-limiting FAO, enzyme carnitine palmitoyltransferase 1 (17). Finally, AMPK activation increases lipolysis, although it can positively or negatively regulate distinct lipolysis enzymes, hormone-sensitive lipase (HSL) and adipose triglyceride lipase (ATGL), in a context-specific manner (18).

Much like transcription and signaling events, metabolites regulate adipocyte differentiation (19). To induce adipogenesis, glucose generates NADPH, a cofactor critical for lipogenesis, through the pentose phosphate pathway (20). Branched-chain amino acid catabolism produces lipogenic acetyl-CoA to boost adipogenesis (21). Branched-chain amino acid catabolism also stimulates PPARγ transcriptional activity, suggesting that metabolic regulation of adipogenesis occurs early in the process of differentiation (22). Alternatively, glutamine oxidation is inhibitory to adipogenesis, although the mechanism remains unclear (23). While it is evident that nutrients modulate adipocyte differentiation, the mechanism by which metabolites engage with the signaling and transcriptional machinery to drive this process is poorly understood. Using metabolic profiling, we found that adipocyte differentiation is associated with altered nucleotide accumulation. Inhibition of purine and pyrimidine biosynthesis prevents lipid storage within adipocytes. Unlike in proliferating cells (24, 25), purine inhibition does not block mTORC1 activation in cells undergoing differentiation. Instead, purine inhibition activates AMPK signaling, a negative regulator of lipogenesis. However, rather than altering AMPK substrate phosphorylation, purine inhibition reduces gene expression of lipid metabolism enzymes. This observation led us to examine whether preventing purine biosynthesis interferes with the transcriptional program that regulates adipocyte differentiation. Indeed, inhibition of purine biosynthesis downregulated PPARγ−C/EBPα expression, a necessary transcriptional program that regulates adipogenesis. Thus, our study suggests that sustained purine biosynthesis is an indispensable pathway in the transcriptional activation of adipogenesis.

## Results

### Nucleotide metabolism is an enriched signature accompanying adipocyte differentiation

**Figure 1:**
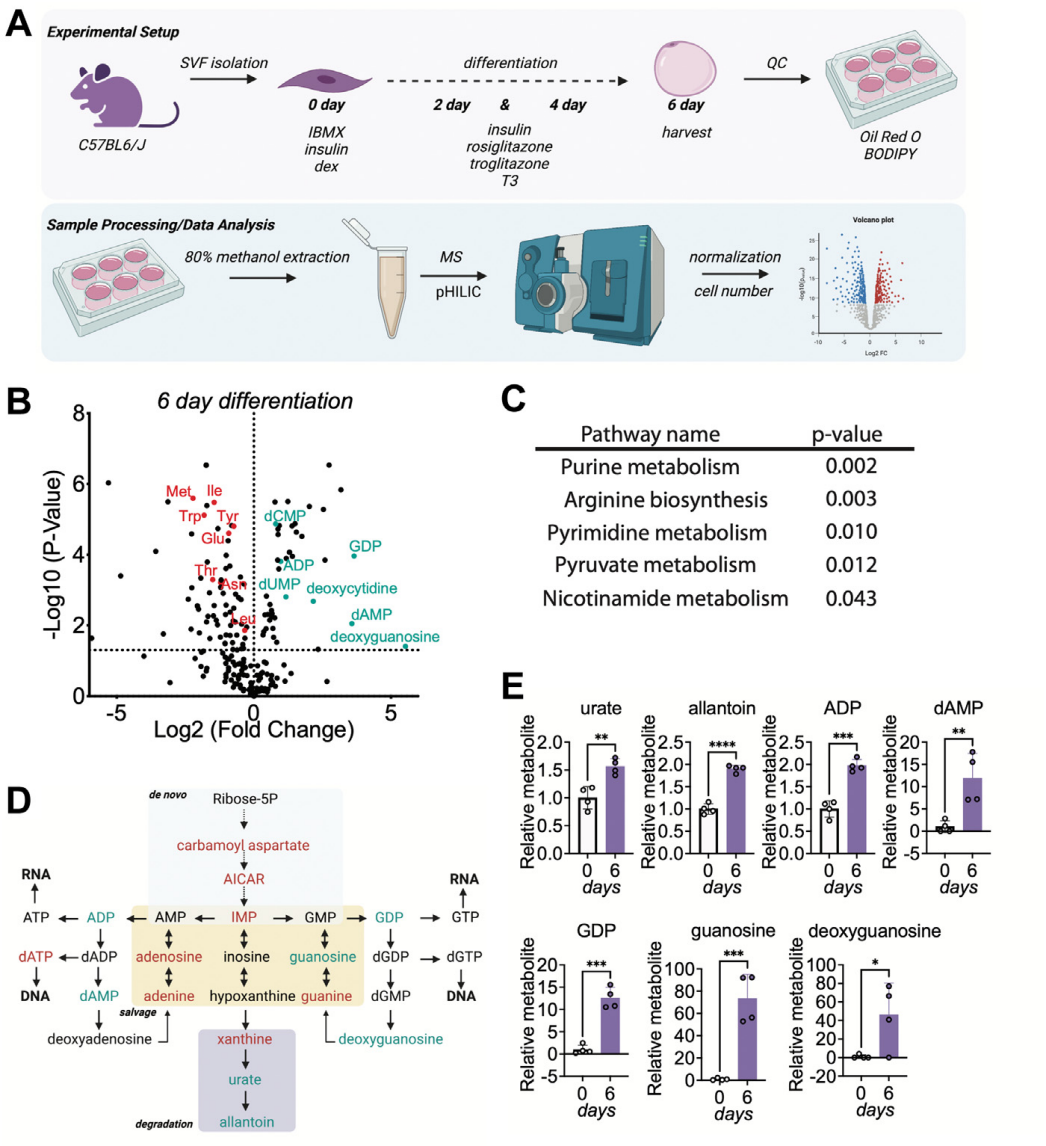
**Nucleotide metabolism is an enriched signature accompanying adipocyte differentiation.***A*, schematic of the experimental setup, the cell model, and sample preparation for steady-state metabolomics. *B*, intracellular abundance of metabolites profiled from 6 days of differentiated primary adipocytes relative to undifferentiated primary preadipocytes. *C*, table of the metabolic pathways significantly affected by 6-day differentiation. The pathway analysis module in MetaboAnalyst 5.0 was used for the analysis. *D*, schematic of the purine biosynthetic pathway illustrating metabolites that are depleted (*red*) or enriched (*green*) in 6-day differentiated primary adipocytes. *E*, relative levels of differentiation-enriched metabolites in the nucleotide biosynthesis pathway. Data shown are from four biological replicates. Statistical significance was determined using the Student’s *t* test. Error bars indicate mean ± SD, ∗*p* ≤ 0.05, ∗∗*p* ≤ 0.01, and ∗∗∗*p* ≤ 0.001.

**Supporting Figure S1:**
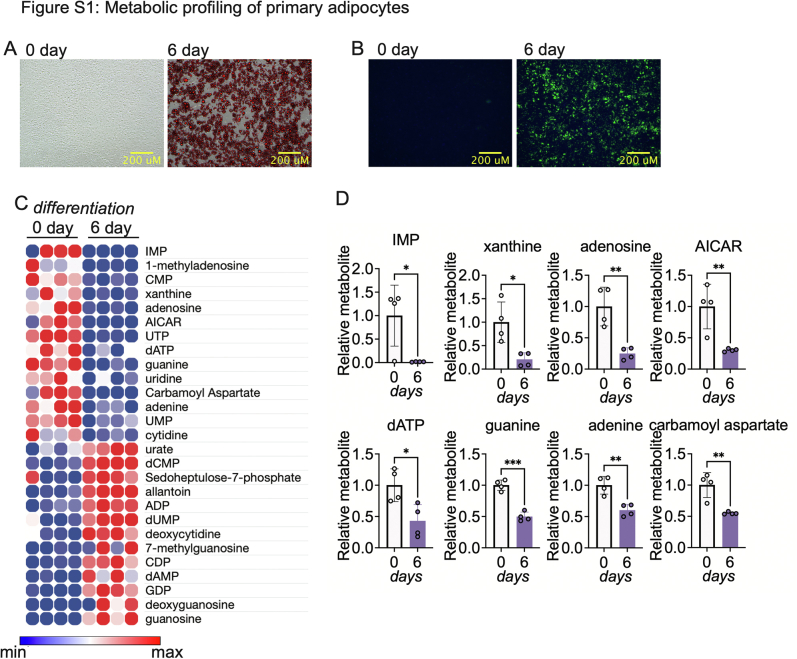
**Nucleotide metabolism is an enriched signature accompanying adipocyte differentiation.***A*, representative images of Oil Red O staining in primary SVF undifferentiated cells and 6-day differentiated adipocytes. *B*, representative images of BODIPY 493/503 staining of primary SVF undifferentiated cells and 6-day differentiated adipocytes. *C*, heat map of metabolites in the nucleotide biosynthesis pathway significantly altered during differentiation. *D*, relative levels of metabolites in the nucleotide metabolism pathway depleted in differentiation. Data shown are from 4 biological replicates. Error bars indicate mean ± SD, ^∗^*p* ≤ 0.05, ^∗∗^*p* ≤ 0.01, and ^∗∗∗^*p* ≤ 0.001. SVF, stromal vascular fraction.

Transcriptional and signaling programs that regulate adipogenesis are well defined. In addition, recent studies have revealed a critical function of metabolic rewiring to support this process (19). However, most of these studies were performed using the immortalized 3T3-L1 system, which originates from a single clone and fails to recapitulate all the characteristics of primary cell culture models (26). We hypothesized that examining metabolic alterations associated with adipogenesis using primary preadipocytes would reveal new metabolic pathways that participate in the initiation and maintenance of this differentiated state and may be relevant *in vivo*. Therefore, primary preadipocytes from the stromal vascular fraction (SVF) were stimulated to differentiate for 6 days using a cocktail containing 3-isobutyl-1-methylxanthine (IBMX), dexamethasone, insulin, rosiglitazone, troglitazone, and triiodothyronine (T3) (Fig. 1*A*). Cell differentiation was confirmed using BODIPY and Oil Red O staining of neutral lipids (Fig. S1, *A* and *B*). Using mass spectrometry, we compared the steady-state metabolite profiles of undifferentiated and 6 day differentiated primary adipocytes (Fig. 1*A*). To gain a broad view of the metabolic rewiring that occurs during differentiation, we profiled a total of 218 metabolites and discovered that 117 metabolites were significantly altered. Our analysis confirmed the depletion of amino acids as previously reported (22), suggesting the conservation of these metabolic pathways during differentiation in primary adipocytes (Fig. 1*B*). Moreover, using MetaboAnalyst 5.0 (https://www.metaboanalyst.ca/MetaboAnalyst/), we observed that the purine and pyrimidine biosynthetic pathways are the top signatures altered during adipocyte differentiation (Fig. 1*C*). Because purine metabolism was the pathway most significantly altered during differentiation, we further focused on examining the relative metabolite levels in this pathway (Fig. S1*C*). Carbamoyl aspartate, 5-aminoimidazole-4-carboxamide ribonucleotide, and inosine monophosphate (IMP), intermediates in the *de novo* purine synthesis pathway required to produce nucleotides, were depleted (Figs. 1*D* and S1, *C* and *D*). ADP and GDP, which are products of the purine synthesis pathway, were generated, suggesting that the nucleotide biosynthetic pathway is engaged during adipogenesis (Figs. 1, *D* and *E* and S1*C*). Urate and allantoin, metabolites in the nucleotide degradation pathway, were similarly enriched in differentiated adipocytes (Figs. 1, *D* and *E* and S1*C*). Metabolites in the nucleotide salvage pathway were both enriched and depleted, suggesting that this pathway is also likely active during adipocyte differentiation (Figs. 1, *D* and *E* and S1*C*). Altogether, our results reveal major alterations in nucleotide abundance associated with adipocyte differentiation.

### Inhibition of nucleotide biosynthesis prevents lipid accumulation in differentiating adipocytes

**Figure 2:**
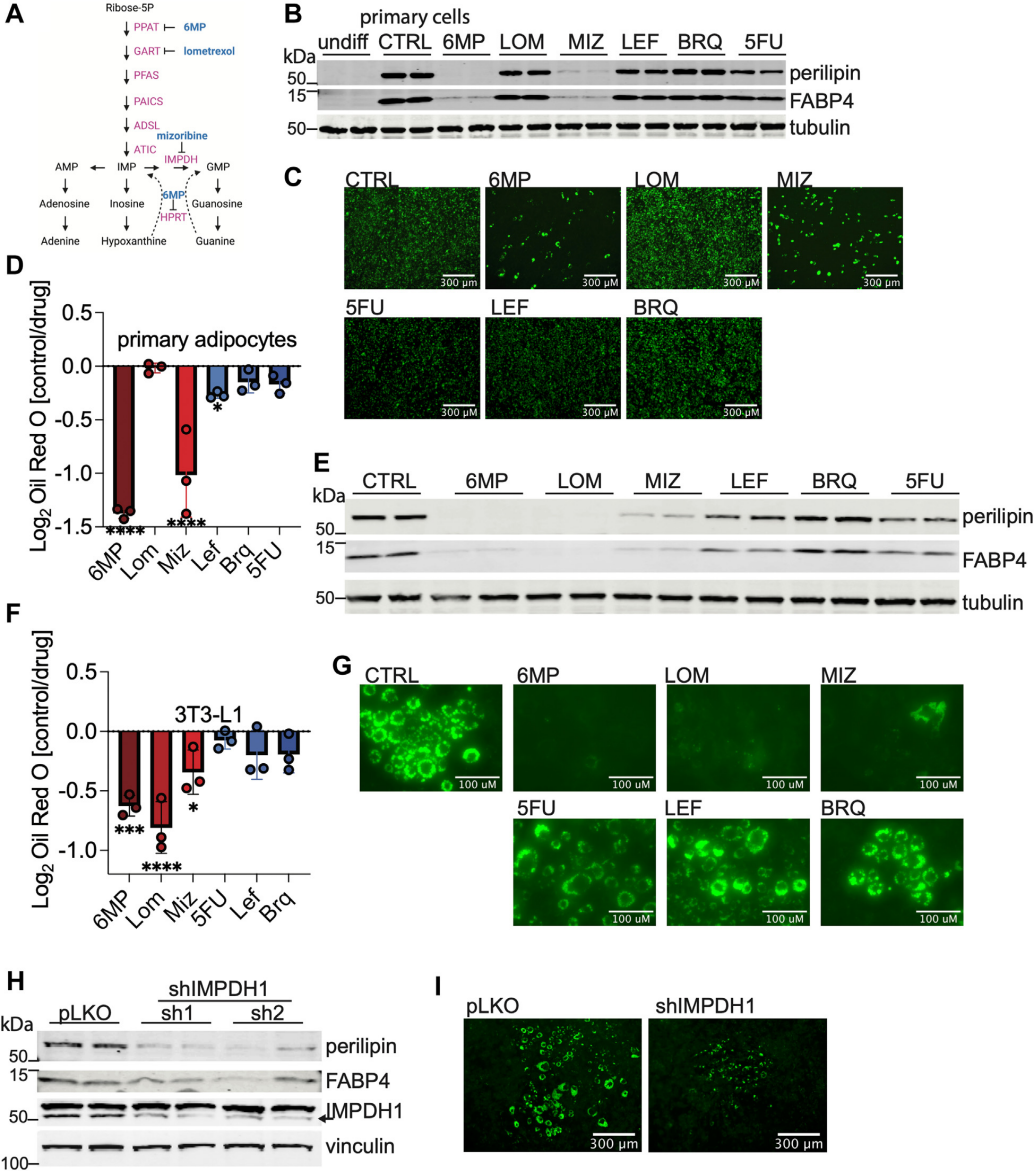
**Inhibition of purine and pyrimidine metabolism disrupts lipid droplets in differentiating adipocytes.***A*, schematic depicting *de novo* purine synthesis and salvage pathways. Inhibitors of purine synthesis pathways in *blue*. *B*, protein levels of perilipin, FABP4, and α-tubulin in undifferentiated SVF preadipocytes or after 6 days of differentiation in the presence or the absence of 50 μM 6-mercaptopurine (6MP), 2 μM lometrexol (LOM), 25 μM mizoribine (MIZ), 10 μM leflunomide (LEF), 1 μM brequinar (BRQ), or 1 μM 5-fluororacil (5FU). Media were changed every 2 days, and drug was replenished. Data shown are from two biological replicates. *C*, representative images of BODIPY 493/503 staining of untreated primary SVF cells or treated with indicated drugs, differentiated for 6 days. *D*, quantification of Oil Red O extracted from primary adipocytes treated with indicated drugs normalized to untreated cells. All cells were differentiated for 6 days. Data shown are from three biological replicates. Statistical significance was determined using one-way ANOVA multiple comparisons test. *E*, protein levels of perilipin, FABP4, and α-tubulin after 6 days of differentiation of 3T3-L1 adipocytes treated as in (*B*). Data shown are from two biological replicates. *F*, quantification of Oil Red O staining in 3T3-L1 adipocytes treated with indicated drugs normalized to untreated cells. All cells differentiated for 6 days. Data shown are from three biological replicates. Statistical significance was determined using one-way ANOVA multiple comparisons test. *G*, representative images of BODIPY 493/503 staining of untreated 3T3-L1 cells or treated with indicated drugs, differentiated for 6 days. *H*, Western blot analysis of perilipin, FABP4, IMPDH1, and tubulin from 3T3-L1 cells infected with shControl or shIMPDH1 (two distinct shRNAs) and differentiated for 8 days. Data shown are from two biological replicates. *I*, representative images of BODIPY 493/503 staining of 3T3-L1 cells infected with shControl or shIMPDH1 and differentiated for 8 days. Error bars indicate mean ± SD, ∗*p* ≤ 0.05, ∗∗*p* ≤ 0.01, and ∗∗∗*p* ≤ 0.001. All data are representative of 2 to 3 independent experiments. FABP4, fatty acid binding protein 4; IMPDH, inosine monophosphate dehydrogenase.

**Supporting Figure S2:**
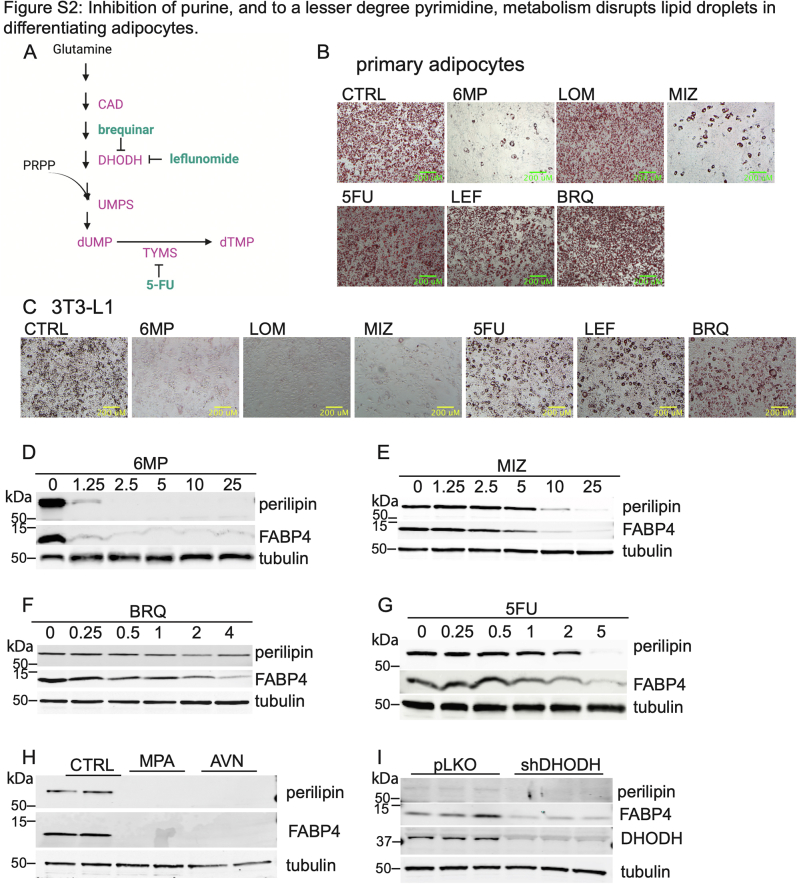
**Inhibition of purine and pyrimidine metabolism disrupts lipid droplets in differentiating adipocytes.***A*, schematic depicting pyrimidine synthesis pathway. Inhibitors of pyrimidine synthesis are shown in green. *B*, Oil Red O staining of primary adipocytes differentiated for 6 days and treated with vehicle or indicated drugs every other day. *C*, Oil Red O staining of 3T3-L1 cells differentiated for 6 days and treated with vehicle or indicated drugs every other day. (*D*) 6MP, (*E*) MIZ, (*F*) BRQ, and (*G*) 5FU dose studies in µM range followed by western blot analysis of perilipin, FABP4, and tubulin. *H*, Western blot analysis of perilipin, FABP4, perilipin, and α-tubulin after 6 days of differentiation in primary adipocytes treated with 10 µM MPA or 10 µM AVN. *I*, Western blot analysis of perilipin, FABP4, DHODH, and tubulin from 3T3-L1 adipocytes infected with shControl or shDHODH and differentiated for 8 days. 6MP, 6-mercaptopurine; MIZ, mizoribine; BRQ, brequinar; 5FU, 5-fluorouracil; FABP4, fatty acid binding protein 4; MPA, mycophenolic acid; AVN, AVN944; DHODH, dihydroorotate dehydrogenase.

To determine the function of nucleotide metabolism in differentiating adipocytes from the SVF, we treated cells with inhibitors of *de novo* purine synthesis or purine salvage and inhibitors of *de novo* pyrimidine biosynthesis while inducing differentiation (Figs. 2*A* and S2*A*) (27, 28). Inhibition of *de novo* purine synthesis enzymes inosine monophosphate dehydrogenase 1 and 2 (IMPDH1 and IIMPDH2) with mizoribine (MIZ) or phosphoribosyl pyrophosphate amidotransferase with 6-mercaptopurine (6MP) resulted in inhibited expression of perilipin and fatty acid binding protein 4 (FABP4), markers of lipid storage and adipocyte differentiation, as measured by Western blot, and decreased lipid accumulation as visualized by Oil Red O and BODIPY in primary adipocytes (Figs. 2, *B*–*D* and S2*B*). Of note, 6MP may also inhibit the purine salvage pathway *via* hypoxanthine–guanine phosphoribosyltransferase 1 and thus may be a more potent inhibitor of adipocyte differentiation. The broader activity of 6MP may explain why this inhibitor exhibits less dose-dependent activity on the expression of adipogenic markers than MIZ (Fig. S2, *D* and *E*). Inhibition of *de novo* purine synthesis enzyme phosphoribosylglycinamide formyltransferase with lometrexol (LOM) had no effect on lipid accumulation in primary adipocytes. Inhibition of pyrimidine synthesis enzymes dihydroorotate dehydrogenase (DHODH) with leflunomide or brequinar (BRQ) or thymidylate synthase with 5-fluorouracil (5FU) had a lesser effect on expression of differentiated state markers and lipid accumulation (Figs. S2, *A* and *B*, and 2, *B*–*D*). However, with increasing drug concentrations, BRQ and 5FU produced an inhibitory effect on adipogenic markers (Fig. S2, *F* and *G*). We next sought to determine whether inhibition of nucleotide biosynthesis also influences differentiation in 3T3-L1 cells, a homogenous preadipocyte population. Blocking purine biosynthesis with LOM, MIZ, or 6MP resulted in a reduced expression of differentiation markers perilipin and FABP4 and decreased lipid accumulation (Figs. 2, *E*–*G* and S2*C*). The distinct effects of LOM on lipid accumulation in primary *versus* 3T3-L1 cells raise the possibility that compensatory mechanisms may counteract the loss of purine biosynthesis in primary cells. To identify whether structurally distinct compounds targeting a single enzyme have comparable effects on lipid accumulation, we examined the effects of IMPDH inhibitors mycophenolic acid (MPA) and AVN944 (AVN). Both MPA and AVN blocked the expression of perilipin and FABP4, mimicking the actions of MIZ (Fig. S2*H*). As in primary adipocytes, inhibition of pyrimidine biosynthesis had a lesser effect on lipid accumulation during 3T3-L1 differentiation (Figs. 2, *E*–*G* and S2*C*). Genetic perturbation of purine synthesis *via* shRNA-mediated knockdown of IMPDH1 had a similar inhibitory effect on 3T3-L1 differentiation, as evidenced by decreased expression of perilipin and FABP4 and decreased BODIPY staining (Fig. 2, *H* and *I*). Similarly, the knockdown of DHODH reduced the expression of perilipin and FABP4, supporting the requirement of pyrimidine biosynthesis in differentiating adipocytes (Fig. S2*I*). Collectively, our results demonstrate that disrupting nucleotide metabolism blocks lipid accumulation in differentiating adipocytes.

### Inhibition of nucleotide metabolism does not obstruct mTORC1 signaling in differentiating adipocytes

**Figure 3:**
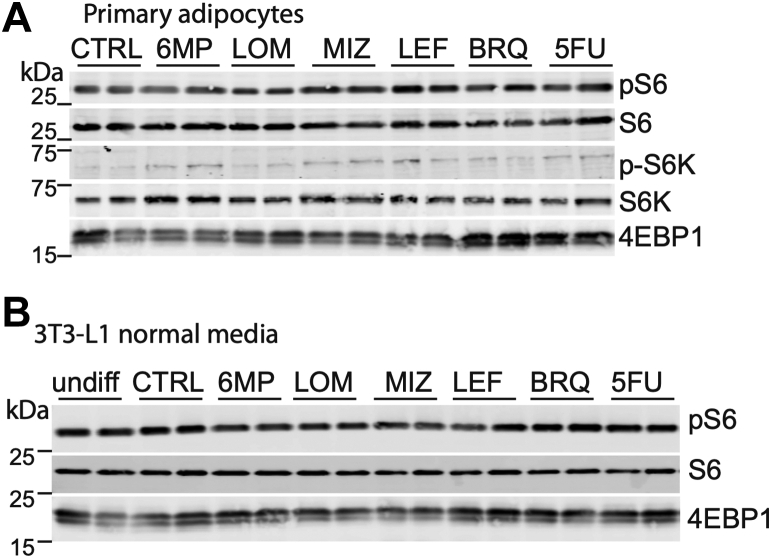
**Inhibition of purine metabolism in differentiating cells does not alter mTORC1 activity.***A*, Western blot analysis of pS6, S6, pS6K, S6K, and 4E-BP1 after 6 days of SVF cell differentiation into primary adipocytes and treatment with indicated drugs as in Figure 2*B*. Data shown are from two biological replicates. *B*, Western blot analysis of pS6, S6, and 4E-BP1 after 6 days of differentiation in 3T3-L1 cells and treatment with indicated drugs. Data shown are from two biological replicates. All data are representative of 2 to 3 independent experiments. mTORC, mechanistic target of rapamycin complex.

**Supporting Figure S3:**
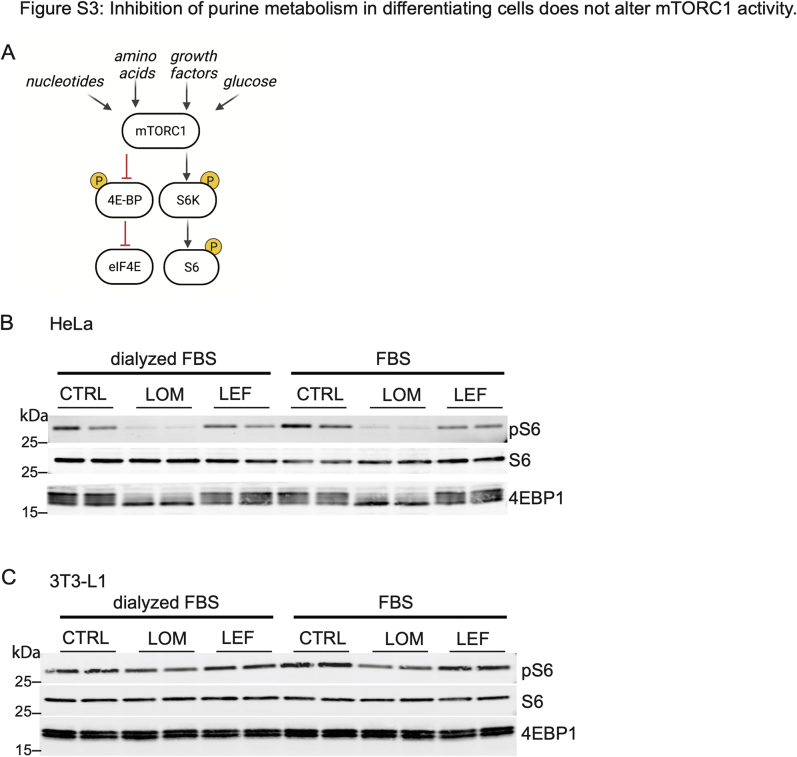
**Inhibition of purine metabolism in differentiating cells does not alter mTORC1 activity.***A*, schematic of mTORC1 substrate phosphorylation. (*B*) Western blot analysis of phospho-S6, S6, and 4E-BP1 in HeLa cells. Cells cultured in DMEM supplemented with 10% FBS or 10% dialyzed FBS and treated with 2 µM LOM and 10 µM LEF for 24 hours. (*C*) Western blot analysis of pS6, S6, and 4E-BP1 in 3T3-L1 cells. Cells cultured in DMEM supplemented with 10% FBS or 10% dialyzed FBS and treated with LOM and LEF as in S3B for 24 hours. All data are representative of 2-3 independent experiments. mTORC1, mechanistic target of rapamycin complex 1; DMEM, Dulbecco’s modified Eagle's medium; FBS, fetal bovine serum; LOM, lometrexol; LEF, leflunomide.

mTORC1 exquisitely senses nutrients to regulate cell growth and stimulate anabolic processes such as lipogenesis (29). As such, purine depletion prevents mTORC1 activation in some proliferating cell types (24, 25). We probed the effect of inhibiting nucleotide biosynthesis on mTORC1 activity by measuring the phosphorylation of its substrates S6 and S6K or mobility shift of 4E-BP1, indicative of a change in phosphorylation (Fig. S3*A*). While we observed the previously reported loss of mTORC1 signaling by blocking purine metabolism in HeLa cells (24, 25), proliferating 3T3-L1 cells were much less sensitive to such sensing, as evidenced by maintained S6 phosphorylation (Fig. S3, *B* and *C*). Moreover, differentiating primary adipocytes or 3T3-L1 cells for 6 days in the presence of purine or pyrimidine metabolism inhibitors did not prevent phosphorylation of mTORC1 substrates S6 or S6K or alter mobility of 4E-BP1 (Fig. 3, *A* and *B*). These data suggest that the disruption of lipid accumulation observed in adipocytes following inhibition of nucleotide biosynthesis is not dependent on mTORC1 inactivation.

### Inhibition of purine biosynthesis activates phosphorylation of AMPK but not its targets in differentiating adipocytes

**Figure 4:**
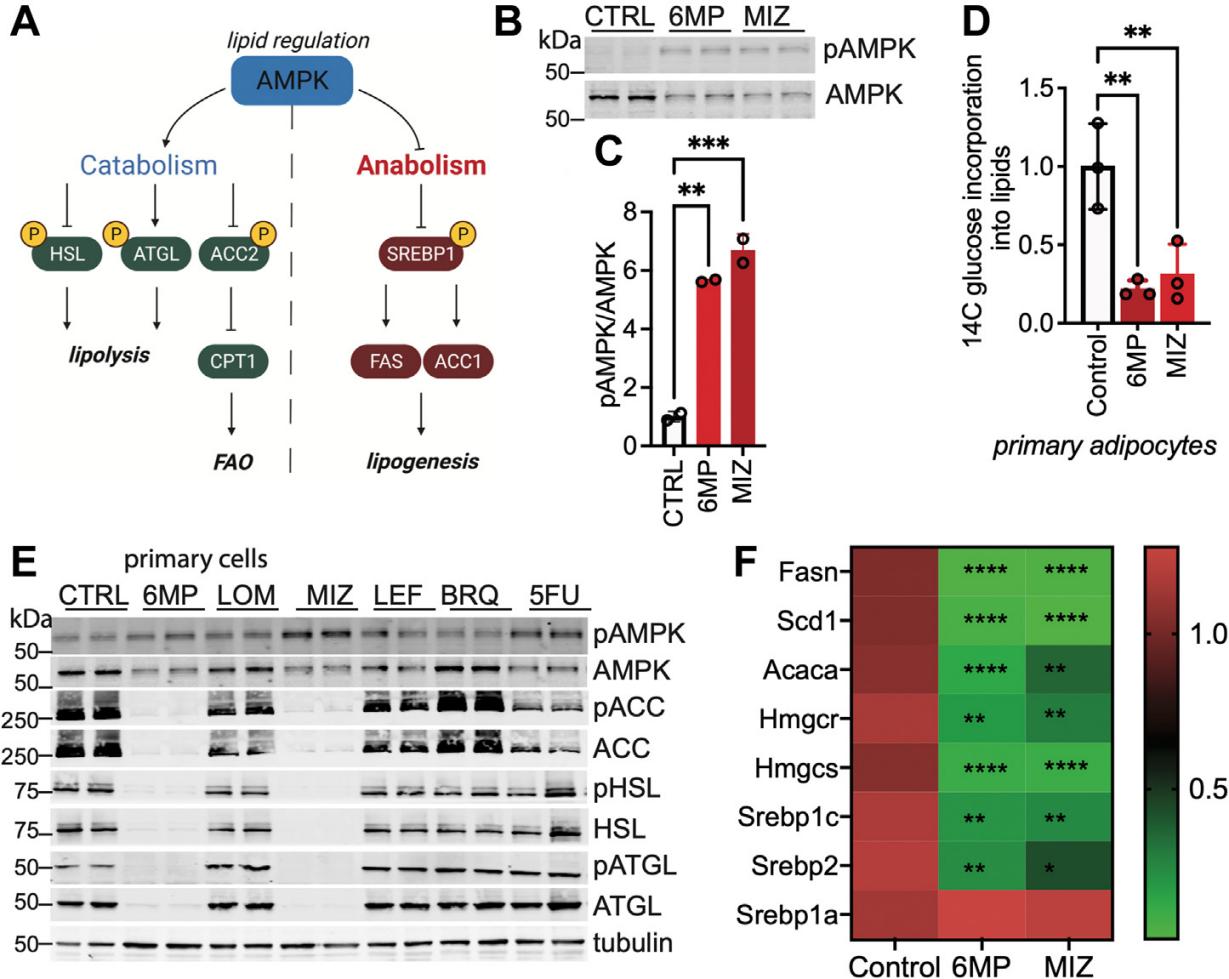
**Inhibition of purine biosynthesis in differentiating cells potentiates AMPK and disrupts lipogenesis.***A*, schematic of AMPK regulation of lipid metabolism. *B*, Western blot analysis of pAMPK and AMPK after 6MP and MIZ treatment in primary SVF cells differentiated into adipocytes for 6 days. Data shown are from two biological replicates. *C*, quantification of band intensity from (*B*). Statistical significance was determined using one-way ANOVA multiple comparisons test. Error bars indicate mean ± SD, ∗∗*p* ≤ 0.01 and ∗∗∗*p* ≤ 0.001. *D*, 1-^14^C glucose incorporation into lipid fraction analyzed from primary SVF cells differentiated for 6 days and treated with vehicle, 6MP, or MIZ. Statistical significance was determined using one-way ANOVA multiple comparisons test. Error bars indicate mean ± SD, ∗∗*p* ≤ 0.01. *E*, Western blot analysis of pAMPK, AMPK, pACC, ACC, pHSL, HSL, pATGL, and ATGL in primary SVF cells after 6 days of differentiation and treatment with indicated drugs. Data shown are from two biological replicates. *F*, gene expression profile in primary SVF cells differentiated for 6 days and treated with vehicle, 6MP, or MIZ. Statistical significance was determined using one-way ANOVA multiple comparisons test. All data are representative of 2 to 3 independent experiments. 6MP, 6-mercaptopurine; ACC, acetyl CoA carboxylase 1; AMPK, AMP-activated protein kinase; ATGL, adipose triglyceride lipase; MIZ, mizoribine.

**Supporting Figure S4:**
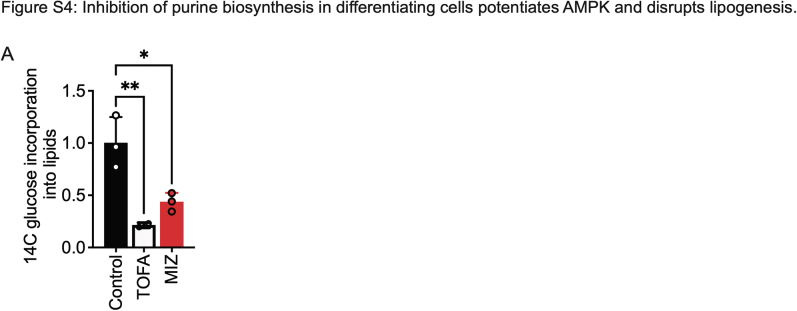
**Inhibition of purine biosynthesis in differentiating cells potentiates AMPK and disrupts lipogenesis.***A*, 1-^14^C glucose incorporation into lipid fraction analyzed from 3T3-L1 adipocytes differentiated for 6 days and treated with vehicle or MIZ. TOFA was added during starvation and during 1-^14^C glucose labeling. Data shown are from 3 biological replicates. Error bars indicate mean ± SD, ^∗^*p* ≤ 0.05, ^∗∗^*p* ≤ 0.01, and ^∗∗∗^*p* ≤ 0.001. Data are representative of 2-3 independent experiments. AMPK, AMP-activated protein kinase; MIZ, mizoribine.

AMPK is another energy and nutrient sensor that regulates lipid metabolism in response to stress (30) (Fig. 4*A*). We probed the effect of inhibiting nucleotide biosynthesis on AMPK by measuring its activating phosphorylation at residue T172. Suppression of purine biosynthesis with 6MP and MIZ results in modest AMPK activation as evidenced by increased phosphorylation (Fig. 4, *B* and *C*). Because AMPK activation blocks lipogenesis (Fig. 4*A*), we sought to investigate whether MIZ and 6MP also affected this functional output. We measured *de novo* lipogenesis by analyzing the incorporation of ^14^C-glucose into the lipid fraction in the presence of an ACC inhibitor, 5-tetradecyloxy-2-furoic acid and detected decreased incorporation, indicating that the assay is adequate to measure lipogenesis (Fig. S4*A*). In MIZ- and 6MP-treated SVF cells differentiated for 6 days, *de novo* lipogenesis is diminished, which is consistent with the decrease in both the transcription and protein expression of enzymes that regulate this process (Fig. 4*D*). We noted a similar effect of inhibition of purine biosynthesis on lipogenesis in 3T3-L1 cells differentiated for 6 days (Fig. S4*A*). AMPK regulates lipid metabolism by directly phosphorylating its substrates, including ACC, HSL, and ATGL (Fig. 4*A*). We examined the effects of purine and pyrimidine biosynthesis inhibitors on the phosphorylation state of AMPK substrates. Inhibiting pyrimidine biosynthesis had no significant effect on the phosphorylation state of AMPK substrates (Fig. 4*E*). Although 6MP and MIZ activate AMPK, we did not observe an increase in phosphorylation of ACC, HSL, and ATGL (Fig. 4*E*). Instead, the expression of these proteins is downregulated by 6MP and MIZ treatment (Fig. 4*E*). Given that mTORC1 remains active following the inhibition of purine biosynthesis in adipocytes, we hypothesized that while protein translation may be functioning properly, transcription may be decreased. Moreover, AMPK is a known regulator of SREBP1 (31), which promotes the transcription of many lipid metabolism enzymes (32, 33, 34). Thus, we examined the mRNA expression of *Srebp* and its targets and found that both MIZ and 6MP downregulate the *Srebp* transcriptional program as well as *Srebp1c* and *Srebp2* expression (Fig. 4*F*). Collectively, our data demonstrate that interfering with purine biosynthesis decreases lipogenesis and final lipid content in differentiating adipocytes. These changes in lipid metabolism may be regulated at the level of transcription.

### Inhibition of purine biosynthesis blocks adipogenesis by downregulating the expression of key transcriptional regulators

**Figure 5:**
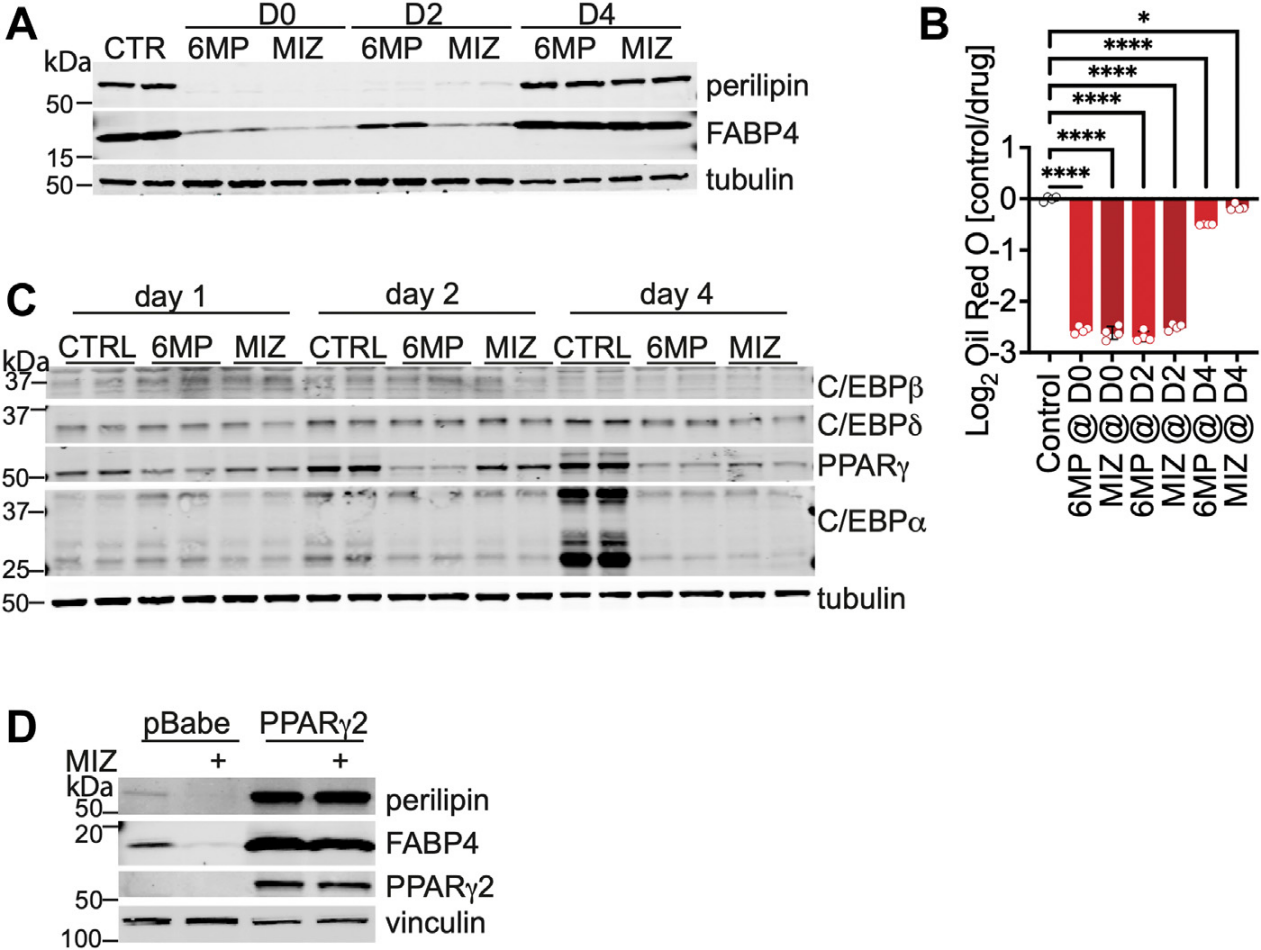
**Inhibition of purine biosynthesis blocks adipogenesis by downregulating the expression of key transcriptional regulators.***A*, Western blotting analysis of primary preadipocytes differentiated for 8 days after adding 6MP or MIZ at day 0 and then every other day, at day 2 and then every other day, or at day 4 and then every other day. Duplicate samples represent two biological replicates. *B*, cells were treated as in (*A*), and Oil Red O staining was performed. Statistical significance was determined using one-way ANOVA multiple comparisons test. Error bars indicate mean ± SD, ∗*p* ≤ 0.05, ∗∗*p* ≤ 0.01, ∗∗∗*p* ≤ 0.001, and ∗∗∗∗*p* ≤ 0.0001. *C*, MIZ and 6MP were added at the start of primary SVF differentiation. Cells were differentiated for 1, 2, or 4 days. C/EBPδ, C/EBPβ, PPARγ, C/EBPα, and tubulin were analyzed by Western blotting. Duplicate samples represent two biological replicates. *D*, 3T3-L1 cells stably expressing pBABE control vector or PPARγ2 were differentiated and treated with 25 μM MIZ or DMSO control for 6 days. Lysates were analyzed by Western blotting as indicated. The data are representative of three independent experiments. 6MP, 6-mercaptopurine; C/EBP, CCAAT/enhancer-binding protein; DMSO, dimethyl sulfoxide; MIZ, mizoribine; PPARγ, peroxisome proliferator–activated receptor γ.

**Supporting Figure S5:**
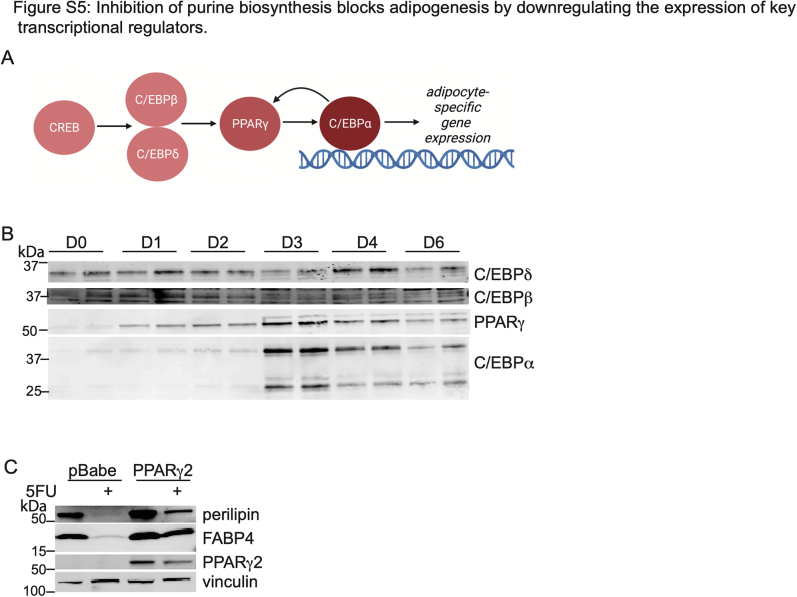
**Inhibition of purine biosynthesis blocks adipogenesis by downregulating the expression of key transcriptional regulators.***A*, model of the transcriptional program that regulates adipogenesis. *B*, primary SVF cells were maintained undifferentiated or differentiated for 1, 2, 3, 4, or 6 days. Equal amount of protein was loaded and C/EBPd, C/EBPb, PPARg, C/EBPa, and tubulin were analyzed by Western blotting. *C*, 3T3-L1 cells stably expressing pBABE control vector or PPARg2 were differentiated and treated with 5 µM 5FU or DMSO control for 6 days. Lysates were analyzed by Western blotting as indicated. The data are representative of 3 independent experiments. SVF, stromal vascular fraction; 5FU, 5-fluorouracil; DMSO, dimethyl sulfoxide.

Given that *Srebp1c* and *Srebp2* were downregulated transcriptionally by purine biosynthesis inhibitors, we postulated that MIZ and 6MP may modulate early events of transcriptional regulation that promote cell differentiation. If our hypothesis is correct, we would detect a lesser or no effect on lipid accumulation if cells are treated with these compounds after transcriptional initiation. Indeed, treating SVF cells with MIZ and 6MP 4 days after initiation of differentiation had minimal effects on lipid accumulation (Fig. 5, *A* and *B*), suggesting that interfering with purine biosynthesis may impair early regulatory events that promote adipogenesis. Blocking purine biosynthesis with 6MP and MIZ 2 days after initiation of differentiation effectively inhibited lipid accumulation and expression of adipogenic markers (Fig. 5, *A* and *B*). To identify the possible factors that may be regulated by inhibition of purine biosynthesis, we profiled transcription factor activation in primary SVF cells stimulated to differentiate into adipocytes over a time course of 6 days (Fig. S5, *A* and *B*). As established in the literature, C/EBPδ and C/EBPβ are activated early and already expressed in preadipocytes, whereas PPARγ and C/EBPα are stimulated after 1 to 3 days of differentiation (Fig. S5, *A* and *B*). Blocking purine biosynthesis did not disrupt the activation of transcriptional regulators that are induced after 1 day of differentiation (Fig. 5*C*) suggesting that CEB/Pδ and C/EBPβ may not be directly regulated by these nucleotides. However, after 2 days of differentiation, expression of PPARγ and C/EBPα was suppressed by both MIZ and 6MP treatment, suggesting that these factors are key transmitters of nucleotide mediation on adipogenesis (Fig. 5*C*). To evaluate whether restoring key transcriptional regulation is sufficient to prevent adipogenic repression induced by the nucleotide biosynthesis inhibitors, we overexpressed PPARγ2. In the context of PPARγ2 overexpression, MIZ and 5FU failed to block adipogenesis as evidenced by maintained expression of perilipin and FABP4 (Figs. 5*D* and S5*C*). Collectively, our data indicate that nucleotide biosynthesis inhibition downregulates transcriptional activators PPARγ and C/EBPα and subsequently blocks adipogenesis.

### Nucleoside and nitrogenous bases rescue the effects of *de novo* purine inhibition on adipogenesis

**Figure 6:**
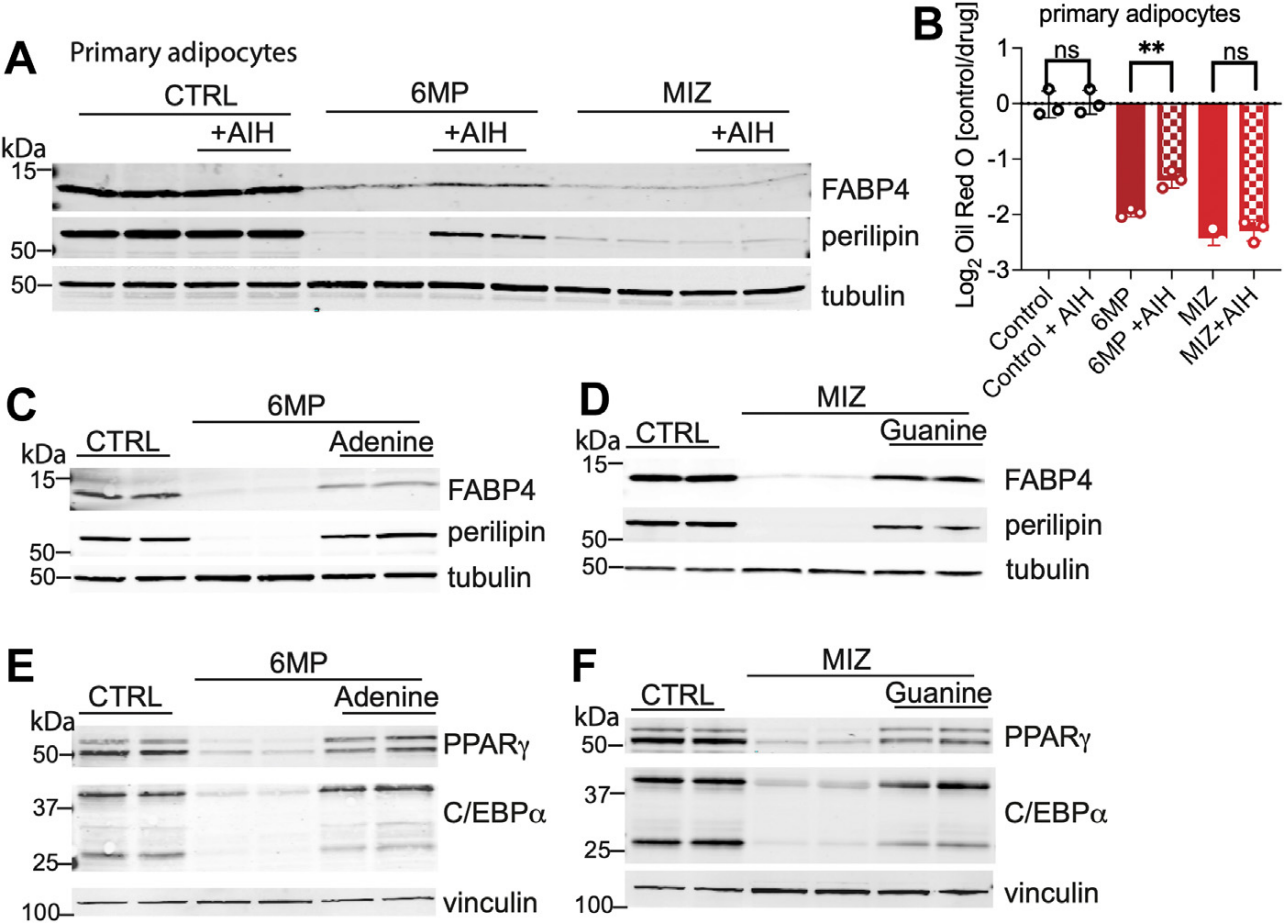
**Nucleoside and nitrogenous bases rescue the effects of *de novo* purine inhibition on adipogenesis.***A*, Western blot analysis of FABP4, perilipin, and α-tubulin after 6 days of differentiation in primary SVF cells treated with 6MP, LOM, or MIZ, and rescued with a cocktail of nucleotides (AIH) containing 5 μM adenosine, 5 μM inosine, and 5 μM hypoxanthine. *B*, quantification of Oil Red O staining in primary SVF cells treated with indicated drugs with or without nucleotide cocktail (as in *A*) normalized to untreated cells, differentiated for 6 days. Statistical significance was determined using one-way ANOVA multiple comparisons test. Error bars indicate mean ± SD, ∗*p* ≤ 0.05, ∗∗*p* ≤ 0.01, and ∗∗∗*p* ≤ 0.001. *C*, Western blot analysis of perilipin, FABP4, and α-tubulin after 6 days of differentiation into primary adipocytes treated with 6MP and rescued with 50 μM adenine. *D*, Western blot analysis of perilipin, FABP4, and α-tubulin after 6 days of differentiation into primary adipocytes treated with MIZ and rescued with 50 μM guanine. *E*, Western blot analysis of PPARγ, C/EBPα, and vinculin after 6 days of differentiation into primary adipocytes treated with 6MP and rescued with 50 μM adenine. *F*, Western blot analysis of PPARγ, C/EBPα, and vinculin after 6 days of differentiation into primary adipocytes treated with MIZ and rescued with 50 μM guanine. Data shown are from two biological replicates. All data are representative of 2 to 3 independent experiments. 6MP, 6-mercaptopurine; C/EBPα, CCAAT/enhancer-binding protein; FABP4, fatty acid binding protein 4; LOM, lometrexol; MIZ, mizoribine; PPARγ, peroxisome proliferator–activated receptor γ.

**Supporting Figure S6:**
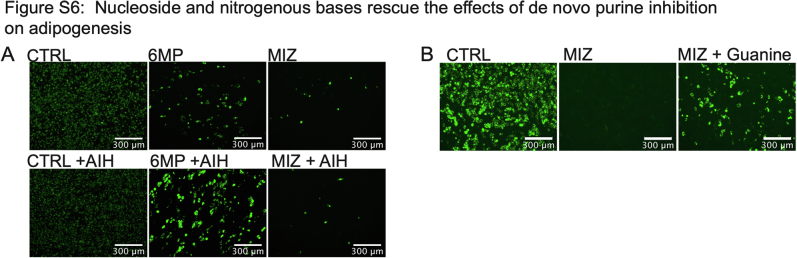
**Nucleoside and nitrogenous bases rescue the effects of *de novo* purine biosynthesis inhibition on adipogenesis.***A*, BODIPY 493/503 staining of primary adipocytes after 6 days of differentiation untreated or treated with 6MP, or 6MP and a nucleoside cocktail (AIH) containing 5 µM adenosine, 5 µM inosine, and 5 µM hypoxanthine. *B*, representative images of BODIPY 493/503 staining after 6 days of differentiation in primary SVF cells that were untreated, treated with MIZ, or MIZ and 50 µM guanine. 6MP, 6-mercaptopurine; MIZ, mizoribine.

We hypothesized that the effects of inhibiting *de novo* purine biosynthesis on adipogenesis may be due to decreased purine availability and thus could be rescued by exogenous nucleoside and nitrogenous bases that can produce nucleotides through the purine salvage pathway. To determine whether adipogenesis can be rescued by the addition of purine nucleosides, we exposed primary SVF cells to adenosine, inosine, and hypoxanthine in the presence of 6MP or MIZ. The addition of nucleosides substantially rescued a 6MP-mediated block in adipogenesis, as assessed by the increased expression of differentiation markers FABP4 and perilipin (Fig. 6*A*), increased Oil Red O accumulation (Fig. 6*B*), and increased BODIPY staining (Fig. S6*A*). MIZ inhibition of adipogenesis was not rescued by adenosine, inosine, and hypoxanthine, as expected, given that MIZ should deplete cellular guanosine and guanine. We next examined whether the addition of nitrogenous bases had the capacity to restore adipogenesis in the presence of purine biosynthesis inhibitors. We observed that adenine addition could rescue the loss of adipogenic markers observed with 6MP (Fig. 6*C*). Similarly, guanine addition rescued the loss of adipogenic markers and decreased lipid accumulation induced by MIZ (Figs. 6*D* and S6*B*). Given that 6MP and MIZ block transcriptional activators of adipogenesis, we next examined whether the addition of nitrogenous bases rescued the expression of PPARγ and C/EBPα (Fig. 6, *E* and *F*). We noted that adenine and guanine addition restored PPARγ and C/EBPα expression, potentially indicating that purines are sensed to modulate transcriptional programs that regulate cellular outcomes, and limitations in purine availability therefore prevent adipocyte differentiation.

## Discussion

In this study, we sought to elucidate how metabolites regulate adipogenesis using a primary SVF adipocyte cell model. Steady-state metabolomics revealed nucleotide metabolism as the topmost signature altered by differentiation. Inhibition of purine, and to a lesser extent pyrimidine, biosynthesis blocks the transcriptional advancement of adipogenesis, decreasing lipid accumulation in both primary and 3T3-L1 adipocytes. Mechanistically, this regulation does not appear to involve mTORC1, which has previously been shown to sense purines and promote purine biosynthesis in proliferating cells (24, 25). Importantly, our studies reveal that the bidirectional regulation between mTORC1 signaling and purine synthesis may not extend to all postmitotic cells.

Because nucleotide availability also alters AMPK activity (35), we focused our studies on this regulator of lipid metabolism. We found that blocking purine biosynthesis increased AMPK phosphorylation. Interestingly, this activation of AMPK did not result in increased phosphorylation of its direct substrates that modulate lipolysis or FAO. Instead, after inhibition of purine biosynthesis, we observed a decreased lipogenic transcriptional profile that may be deactivated through the loss of expression of AMPK substrate SREBP1. Subsequently, our *de novo* lipogenesis assay confirmed the hypothesis that purine inhibitors block lipogenesis in differentiating adipocytes. Our findings agree with previously published work that demonstrated that 5-aminoimidazole-4-carboxamide ribonucleotide, an activator of AMPK, decreases PPARγ expression and differentiation in 3T3-L1 cells (13). Moreover, it has been observed that in multipotent mesenchymal stem cells, activation of AMPK promotes commitment to the osteogenic lineage, whereas suppression of AMPK activity promotes adipogenesis (36). Therefore, future studies may investigate whether antagonizing purine biosynthesis could likewise push pluripotent cells toward the osteogenic lineage.

While we demonstrate that impeding purine biosynthesis dampens the PPARγ-C/EBPα transcriptional profile that is required to drive adipogenesis, the mechanism by which this occurs remains unclear. Significantly, the effect of purine biosynthesis inhibition can be rescued by adding exogenous nucleoside and nitrogenous bases. Previous studies identified xanthine oxidoreductase, an enzyme that catalyzes the catabolism of purines, as a novel regulator of adipogenesis (37). It was demonstrated that xanthine oxidoreductase potentiates PPARγ activation, complementing our findings that altering purine biosynthesis obstructs PPARγ activation. How purine biosynthesis modulates transcriptional regulation remains to be identified. IBMX, a xanthine derivate, is commonly used in adipogenic differentiation cocktails and is thought to promote the process by elevating cAMP and cGMP levels (38). Therefore, one possibility is that purine biosynthesis promotes adipocyte differentiation by increasing cAMP or cGMP availability.

Although nucleotide biosynthesis has been well studied in the context of proliferation, in part because of their importance in DNA and RNA synthesis (27), little is known about the role of nucleotides in cell physiology and cell fate decision. Recent studies reveal that although nucleotide biosynthesis inhibition limits proliferation, it stimulates cell migration and the epithelial–mesenchymal transition transcriptional program characterized by N-cadherin and vimentin upregulation (39). Furthermore, perturbing nucleotide abundance regulates differentiation in various cell systems; depletion of nucleotides stimulates acute myeloid leukemia differentiation, but elevation in nucleotides may also promote cardiac mesoderm lineage through paracrine signaling (40, 41). Now our study adds a new role of nucleotide biosynthesis in the regulation of adipogenesis. Collectively, these studies reveal the critical impact of nucleotide alterations in cell physiology and cell functions beyond proliferation.

In sum, we have identified purine biosynthesis as a required pathway to stimulate adipogenesis. Further studies are warranted to determine whether modulating nucleotide pools can alter adipogenesis and weight gain *in vivo* in the context of overnutrition.

## Experimental procedures

### Primary preadipocyte isolation

Primary SVF cells were isolated from the inguinal white adipose tissue of 4- to 6-day-old mice. The experimental procedures have been approved by the Vanderbilt University Subcommittee on Animal Research Care (IACUC, Institutional Animal Care and Use Committee) as required by the Public Health Service Policy on Humane Care and Use of Laboratory Animals. White adipose tissue was dissected and digested in 1 mg/ml collagenase type II (Sigma; catalog no.: C6885) dissolved in 3% bovine serum albumin (Sigma; catalog no.: A1470) in Hanks buffered saline solution with calcium and magnesium for 30 min at 37 °C while shaking at 300 RPM. Dulbecco’s modified Eagle's medium (DMEM) with high glucose and no sodium pyruvate was supplemented with 10% fetal bovine serum (FBS), 10 μM nonessential amino acids (Thermo; catalog no.: 11140050), 2 mM glutamine, 20 mM Hepes, and 0.1 μM mercaptoethanol (Sigma; catalog no.: M3148) was used for cell washing and separation through a 100 μm cell strainer. The filtered cell suspension was centrifuged at 600*g* for 5 min at 4 °C. The cell pellet was resuspended in the same media and plated. Growth medium was changed every other day until cells reached 100% confluency, at which point differentiation was induced.

### Primary preadipocyte differentiation

DMEM/nutrient mixture F-12 supplemented with 10% FBS, 1% penicillin and streptomycin, 1.7 μM insulin, 1 μM dexamethasone, and 0.5 mM IBMX was used to induce differentiation. Cells were kept in an induction medium for 2 days and then switched to “maintenance media” consisting of DMEM/nutrient mixture F-12 with 10% FBS, 1% penicillin and streptomycin, 17 nM insulin, 2 μM troglitazone, 1 μM rosiglitazone, and 1 nM T3. Maintenance medium was changed every other day until the end point assays.

### 3T3-L1 cell culture

3T3-L1 preadipocytes were purchased from American Type Culture Collection (CL-173). Cells were cultured in DMEM (Corning) supplemented with 10% FBS (Gibco) and 1% penicillin and streptomycin (Gibco). Once confluent, 3T3-L1 cells were stimulated to differentiate with DMEM containing 10% FBS, 1% penicillin, and streptomycin, and a chemical cocktail of 0.5 mM IBMX, 1 μM dexamethasone, and 1.5 μg/ml insulin. Media were changed every 2 days during the differentiation time course.

### Drug treatment

Cells were treated during the differentiation time course as indicated with 25 μM MIZ (Cayman; catalog no.: 23128), 2 μM LOM (Cayman; catalog no.: 18049), 50 μM 6MP (Cayman; catalog no.: 23675), 1 μM 5FU (Cayman; catalog no.: 14416), 1 μM BRQ (Cayman; catalog no.: 24445), 10 μM leflunomide (Cayman; catalog no.: 14860), 10 μM MPA (Cayman; catalog no.: 21716), or 10 μM AVN (Cayman; catalog no.: 21284), unless indicated otherwise in the figure legends.

### Metabolomics

Cells were plated in triplicates for metabolite extraction and in triplicates for cell count normalization. Prior to experiments, cells were differentiated/treated as indicated. For metabolite extraction, cells were washed twice with ice-cold PBS, and polar metabolites were extracted directly on the dish using 1 ml ice-cold LC–MS grade 80:20 methanol:water (Thermo Fisher Scientific). Plates were scraped on dry ice, and lysates were collected in Eppendorf tubes. Lysates were vortexed for 10 min at 4 °C and centrifuged at 16,000*g* for 10 min at 4 °C. Supernatants were immediately dried down in a Vacufuge plus Benchtop Vacuum Concentrator. Dried pellets were stored at −80 °C until they were run on LC–MS.

### LC–MS (polar)

A QExactive bench top orbitrap mass spectrometer equipped with an Ion Max source and a HESI II probe coupled to a Dionex UltiMate 3000 HPLC system (Thermo Fisher Scientific) was used to perform all LC–MS experiments. The instrument underwent mass calibration using the standard calibration mixture every 7 days. About 2 μl of resuspended polar metabolite samples were injected onto a SeQuant ZIC-pHILIC 5 μm 150 × 2.1 mm analytical column equipped with a 2.1 × 20 mm guard column (MilliporeSigma). The column oven was held at 25 °C, and the autosampler tray was held at 4 °C. Buffer A comprised of 20 mM ammonium carbonate and 0.1% ammonium hydroxide. Buffer B was 100% acetonitrile. The chromatographic gradient was run at a flow rate of 0.150 ml/min as follows: 0 to 20 min: linear gradient from 80 to 20% B; 20 to 20.5 min: linear gradient from 20 to 80% B; 20.5 to 28 min: hold at 80% B. The mass spectrometer was operated in full-scan polarity-switching mode, with the spray voltage set to 3.0 kV, the heated capillary at 275 °C, and the HESI probe at 350 °C. The sheath gas flow was 40 units, the auxiliary gas flow was 15 units, and the sweep gas flow was 1 unit. MS data were collected in a range of *m/z* = 70 to 1000. The resolution was set at 70,000, the automatic gain control target at 1 × 10^6^, and the maximum injection time was set at 20 ms. An additional scan (*m/z* = 220–700) was included in negative mode only to enhance the detection of nucleotides.

### Oil Red O staining and quantification

Accumulation of lipids after 6 days of differentiation was assessed by Oil Red O staining. Oil Red O (Sigma) stock solution was prepared as a 0.3% solution in isopropanol. Cells were washed with PBS, fixed with 4% paraformaldehyde for at least 2 h, washed with 60% isopropanol, and then stained with filtered Oil Red O solution (75% Oil Red O stock solution, 25% water). Cells were washed with dH_2_O to remove the excess stain before imaging. Following imaging, Oil Red O stain taken up by lipid droplets was solubilized in 100% isopropanol and quantified by reading absorbance at 492 nm.

### Generation of stable cell lines

pBABE puro PPARγ2 plasmid was obtained from Addgene (#8859). shRNAs against IMPDH1 and DHODH were subcloned in the pLKO.1 puro vector (Addgene Plasmid #8453) at EcoRI and AgeI sites. Primer sequences for cloning were used: sh_DHODH:

Forward: CCGGCGACGGACTGATCATCACAAACTCGAGTTTGTGATGATCAGTCCGTCGTTTTTG,

Reverse: AATTCAAAAACGACGGACTGATCATCACAAACTCGAGTTTGTGATGATCAGTCCGTCG and shIMPDH1_1:

Forward: CCGGGATAAGGTGAAGATCGCACAACTCGAGTTGTGCGATCTTCACCTTATCTTTTTG.

Reverse: AATTCAAAAAGATAAGGTGAAGATCGCACAACTCGAGTTGTGCGATCTTCACCTTATC shIMPDH1_2:

Forward: CCGGCTCCAGAACTAAGTGGTCCATCTCGAGATGGACCACTTAGTTCTGGAGTTTTTG.

Reverse: AATTCAAAAACTCCAGAACTAAGTGGTCCATCTCGAGATGGACCACTTAGTTCTGGAG. Subcloning was confirmed with sequencing. Subcloned plasmids were transfected into human embryonic kidney 293T cells with lentiviral packaging vectors. After 48 h, lentivirus was harvested, and target cells were infected in the presence of 10 mg/ml polybrene. Following infection, cells were selected with puromycin.

### BODIPY staining and imaging

Accumulation of lipids after 6 days of differentiation was measured by BODIPY staining in live cells. Media were replaced with 500 μl DMEM containing 10% FBS and 1% penicillin and streptomycin. BODIPY 493/503 (Cayman) was prepared to a working concentration of 1:500 in DMEM without serum or antibiotics. About 500 μl of this solution was added to the cells and incubated for 30 min at 37 °C. Cells were imaged using a fluorescence microscope (Evos M5000; Life Technologies).

### Western blotting

Adherent cells were washed twice with PBS and lysed with radioimmunoprecipitation assay lysis buffer (1% NP-40, 150 mM NaCl, 25 mM Tris base, 0.5% sodium deoxycholate, 0.1% SDS, 1% phosphatase inhibitor cocktails #2 and #3 [Sigma], one cOmplete protease inhibitor tablet [Sigma]). Protein content was quantified using a Bicinchoninic Acid assay (Thermo Scientific), and equal protein was run on 4 to 20% Tris–Glycine Gels (Invitrogen). Protein was transferred to a nitrocellulose membrane (Bio-Rad). Membranes were incubated with primary antibodies overnight at 4 °C: perilipin (CST; catalog no.: 9349), FABP4 (CST; catalog no.: 2120), α-tubulin (Novus; catalog no.: NB100-690), phospho-S6 240/244 (CST; catalog no.: 5364), RPS6 (Novus; catalog no.: NB100-1595), phospho-p70 Thr389 (CST; catalog no.: 9234), p70 49D7 (CST; catalog no.: 2708), phospho-Acetyl-CoA Carboxylase (CST; catalog no.: 3661), acetyl-CoA carboxylase (CST; catalog no.: 3662), phospho-AMPKα (CST; catalog no.: 2535), AMPKα (Invitrogen; catalog no.: MA5-15815), 4EBP1 (CST; catalog no.: 9644), p-HSL Ser565 (CST; catalog no.: 4137T), HSL (CST; catalog no.: 4107T), ATGL (CST; catalog no.: 2439S), p-ATGL (Abcam; catalog no.: ab135093), C/EBPδ (CST; catalog no.: 2318T), C/EBPβ (CST; catalog no.: 3087S), PPARγ (CST; catalog no.: 2443S), and C/EBPα (CST; catalog no.: 8178S). Secondary antibodies used were at 1:10,000: IRDye 800CW Donkey Antimouse immunoglobulin G (H + L) (Li-Cor; catalog no.: 925-32212) and IRDye 680RD Donkey Anti-Rabbit immunoglobulin G (H + L) (Li-Cor; catalog no.: 926-68073). Blots were imaged with the Li-Cor Odyssey CLx infrared imaging system and are representative of at least two independent experiments.

### RNA isolation and RT–PCR

RNA was extracted with the Quick-RNA MiniPrep kit (Zymo Research) directly from adherent cells. Complementary DNA was synthesized from 1 μg of RNA using the iScript complementary DNA synthesis kit (Bio-Rad). Real-time quantitative PCR was performed on a Bio-Rad CFX96 using SsoAdvanced Universal SYBR Green SuperMix (Bio-Rad). Mouse quantitative PCR primer sequences used are listed:

Fasn: Forward: CAGCAGAGTCTACAGCTACCT and Reverse: AACACCAGAGACCGTTATGC;

Scd1: Forward: GAAGTCCACGCTCGATCTCA and Reverse: TGGAGATCTCTTGGAGCATGTG;

Acaca: Forward: TGACAGACTGATCGCAGAGAAAG and Reverse: TGGAGAGCCCCACACACA;

Hmgcr: Forward: CTTGTGGAATGCCTTGTGATTG and Reverse: AGCCGAAGCAGCACATGAT;

Hmgcs: Forward: GCCGTGAACTGGGTCGAA and Reverse: GCATATATAGCAATGTCTCCTGCAA;

Srebp1c: Forward: GGAGCCATGGATTGCACATT and Reverse: GGCCCGGGAAGTCACTGT;

Srebp1a: Forward: GGCCGAGATGTGCGAACT and Reverse: TTGTTGATGAGCTGGAGCATGT;

Srebp2: Forward: GCGTTCTGGAGACCATGGA and Reverse: ACAAAGTTGCTCTGAAAACAAATCA.

### Lipogenesis

For measurement of lipogenesis, cells were starved in no-glucose serum-free media for 24 h. Following starvation, labeling with 1-^14^C glucose (PerkinElmer) was performed overnight. Cells were washed twice with PBS before lysis in 0.5% Triton X-100. The lipid fraction was extracted by the addition of chloroform and methanol (2:1 v/v). Samples were centrifuged, and ^14^C incorporation was measured from the lipid-containing phase using a scintillation counter. Each condition was normalized to total cellular protein concentrations and assessed using a Bicinchoninic Acid Protein Assay Kit (ThermoFisher Scientific).

### Quantification and statistical analysis

Details regarding the specific statistical tests, the definition of center, and the number of replicates (n) can be found for each experiment in the figure legends. GraphPad Prism (GraphPad Software, Inc) and MS Excel were used for all quantifications and statistical analyses.

## Data availability

Any information required to reanalyze the data reported in this article is available from the lead contact upon request.

## Supporting information

This article contains supporting information.

## Conflict of interest

The authors declare that they have no conflicts of interest with the contents of this article.
